# Zinc Oxide Coating Effect for the Dye Removal and Photocatalytic Mechanisms of Flower-Like MoS_2_ Nanoparticles

**DOI:** 10.1186/s11671-017-2005-0

**Published:** 2017-03-23

**Authors:** Qingyong Tian, Wei Wu, Shuanglei Yang, Jun Liu, Weijing Yao, Feng Ren, Changzhong Jiang

**Affiliations:** 1 School of Printing and Packaging and School of Physics and Technology, Wuhan University, Wuhan, 430072 People’s Republic of China; 2 Suzhou Research Institute of Wuhan University, Suzhou, 215000 People’s Republic of China

**Keywords:** Molybdenum disulfide, Zinc oxide, Absorbance, Photocatalyst, *p-n* heterostructure

## Abstract

Flower-like MoS_2_ nanoparticles (NPs) consist of ultra-thin MoS_2_ nanosheets are synthesized via a facile one-pot hydrothermal method. The MoS_2_/ZnO *p-n* heterostructure is formed by coating *n*-type ZnO on the surface of flower-like MoS_2_ NPs through the seed-mediate route and post-annealing treatment. The effects for the dye removal and photocatalytic performances after ZnO coating are systematically investigated. The results demonstrated that the coating of ZnO nanoparticles has a positive promotion to the photodegrading properties while negative effect on the adsorption capacity of the MoS_2_/ZnO heterostructures. The related mechanisms on the relationship of adsorption capacity and photocatalysis are discussed in detail.

**Electronic supplementary material:**

The online version of this article (doi:10.1186/s11671-017-2005-0) contains supplementary material, which is available to authorized users.

## Background

Synthetic dyes are massively produced and widely used in many areas nowadays, while most of them are the hazardous materials in the wastewater. Because they are environmentally harmful and not quite prone to degradation, the daily life and health of human beings are imperceptibly influenced by these synthetic dyes [1]. Therefore, the development of efficient methodology or technology to decompose these synthetic dyes becomes emergency issues for environment remediation. Various reports demonstrated that adsorption and photocatalytic degradation are the conventional, effective, and economical methodologies in wastewater purification and environmental protection over the past decades [2–4].

From a technical point of view, semiconductor-based photocatalysts with excellent photoelectric properties have always been the candidates for environmental remediation and clean energy, including metallic oxides (TiO_2_, ZnO, Fe_2_O_3_) [5–10], metallic sulfides (CdS, MoS_2_, CdSe) [11–14], and metallic phosphides (InP, Ni_2_P) [15, 16]. Among those photocatalysts, the two-dimensional (2D) transition-metal dichalcogenides (TMDs) have received an increasing attention for the strong anisotropy of their electrical, chemical, mechanical and thermal properties. Molybdenum disulfide (MoS_2_) is one of the most studied examples in recent years [17–19]. Interestingly, MoS_2_ has enormous potential application value in the field of low-cost adsorbents and environmental friendly photocatalysts because of its tunable band gap (1.29–1.9 eV) and morphology [20].

In fact, the MoS_2_-based composite photocatalysts that are coupled with TiO_2_, ZnO, CdS, or reduced graphene oxide (RGO) have been reported recently [11, 21–24]. For example, the P-doped 2D-2D ZnO/MoS_2_ nanocomposites show excellent photocatalytic activity in methyl blue degradation [22]. The P-doped ZnO nanosheets were prepared through conventional chemical vapor transportation and condensation (CVTC) method, and the 2D MoS_2_ was prepared via liquid exfoliation method, then the ZnO nanosheets/MoS_2_ hybrid photocatalysts were formed by ultrasonically handling and magnetically stirring. This was a complicated preparation process, and the ZnO and MoS_2_ will be in loose contact with each other only by physically mixing, which is not beneficial for the efficient transfer of energy and carriers. In addition, most of the previous studies on MoS_2_-based composite photocatalysts reveal that the enhanced photocatalytic performance could be attributed to the synergetic effect of effective light-response, *p-n* heterojunctions, and large surface areas. However, there are scarcely researches that focused on the influence of the coating effect to the relationship between adsorption capacity and photocatalysts. Indeed, the adsorption and desorption equilibrium between the dyes and the photocatalysts before light illumination show positive promotion effect on the photodegradation process. It has been reported that strong surface adsorption performance is beneficial for the effective degradation of organic dyes during the photocatalytic reaction [25, 26]. For example, the BiOI microspheres have more effective surface adsorption than random BiOI nanoplatelets, which lead to more excellent performance for the degradation of tetracycline hydrochloride (TC) [27].

Therefore, the relationship of adsorption and photocatalysts need to be understood before the application. Good adsorption ability means the organic dyes tend to be in closer contact with the photocatalysts, which is beneficial for the transmission of photogenerated carriers from photocatalysts to organic dye molecules. Strong surface adsorption ability portends more active sites for the photodegrading reaction as well [3]. Moreover, different components will lead to dual removal mechanisms between surface adsorption and photocatalytic degradation in the process of organic dye removal. For instance, the composite photocatalysts combining TiO_2_ with highly adsorptive multi-walled carbon nanotubes (MWCNT) demonstrated high removal rate of methyl orange (MO) dyes in aqueous solutions [28].

Herein, we prepared the MoS_2_/ZnO heterostructures by coating the tunable ZnO layers onto the surface of flower-like MoS_2_ nanoparticles with facile methods. The adsorption and photocatalytic abilities of MoS_2_ NPs have been investigated before and after ZnO coating. The adsorption results illustrate MoS_2_ is the main contributor to the adsorption capacity, which is non-selective adsorption to the organic dyes. After ZnO coating, the adsorption capacity is decreasing and the photocatalytic ability is increasing. And hence the relationship between the surface adsorption and photocatalytic degradation was discussed in detail.

## Methods

### Materials and Chemicals

Sodium molybdate dehydrate (Na_2_MoO_4_ · 2H_2_O), thiourea (CS(NH_2_)_2_), and ethanol (C_2_H_5_OH) were purchased from Sinopharm Chemical Reagent Co., Ltd. Ammonia (NH_3_ · H_2_O, 25%) and zinc acetate (Zn(CH_3_COO)_2_), Rhodamine B (RhB), methyl blue (MB), Rhodamine 6G (Rh6G), and methyl orange (MO) were purchased from Shanghai Jingchun Chemical Reagent Co., Ltd. All the used reagents were analytically pure (AR) without any further purification.

### Synthesis of MoS_2_ Nanoparticles

In a typical process, 93.75 mg of Na_2_MoO_4_ · 2H_2_O and 185 mg of thiourea was dispersed into 30 ml of H_2_O by rigorous stirring for 5 min. Then, 1 ml of concentrated hydrochloric acid (35% wt.) was added into the solution with stirring to form a homogenous mixture. The mixture was transferred into a 50-ml Teflon-lined autoclave after another 5 min continuous stirring. Finally, the sealed autoclave was placed into the oven and kept for 24 h at 200 °C. The precipitate was centrifuged and sequentially washed by H_2_O for several times. The as-synthesized products were dried (60 °C, 12 h) for further measurement.

### Synthesis of MoS_2_/ZnO Heterostructural Photocatalyst

Fifteen milligrams of as-synthesized MoS_2_ nanoparticles were dispersed in a flask containing 22.5 ml of H_2_O under stirring. Then, 12.5 ml of Zn(CH_3_COO)_2_ solution with certain concentration (0.01, 0.02, 0.03, 0.05, and 0.1 M) were added into the mixture and subsequently heated to 40 °C. And then 4 ml of ammonia (5% wt) was added dropwise into the above mixture. After 1 h, the mixture was centrifuged at 5000 rpm. The precipitate was washed to remove the CH_3_COO^−^ and dried, and the final as-obtained powders were annealed under vacuum at 200 °C for 2 h. The theoretical mass proportion can be calculated as MoS_2_/ZnO (3:2), MoS_2_/ZnO (3:4), MoS_2_/ZnO (3:6), MoS_2_/ZnO (3:10), and MoS_2_/ZnO (3:20).

### Adsorption and Photocatalytic Degradation Test

For the adsorption tests, 10 mL of organic dyes (RhB, MB, MO, and Rh6G) in aqueous solution with different concentrations were added into the quartz tube with 5 mg of the as-prepared samples. The suspension was homogenized by sonication and placed in the dark environment with continuous vigorous stirring. The adsorption/desorption equilibrium curves were measured at 5-min intervals by the UV-vis spectra on the Shimadzu UV-2550 spectrophotometer. The photocatalytic performances of MoS_2_/ZnO heterostructures were characterized by investigating the degradation of RhB with 10 mg/L. The preliminary homogenized solutions were illuminated under the simulated sunlight environment after reaching the adsorption/desorption equilibrium. The light source is mercury and tungsten mixed light lamp (300 W). And the corresponding UV-vis spectra were measured at 15 min intervals.

### Characterization

Scanning electron microscopy (SEM) was carried out on a FEI Nova 400 NanoSEM at accelerating voltage of 20 kV. Transmission electron microscopy (TEM), high-resolution TEM (HRTEM) images, and energy-dispersive X-ray spectroscopy (EDX) were performed on a JEOL JEM-2100F transmission electron microscope at 200 kV. The X-ray diffraction (XRD) patterns were recorded in a PANalytical X'Pert PRO X-ray diffractometer with Co Ka radiation. X-ray photoelectron spectroscopy (XPS) analysis was performed on a Thermo Fisher ESCALAB 250Xi system equipped with a monochromatic Al Ka (1486.6 eV) as the radiation source.

## Results and Discussion

**Fig. 1:**
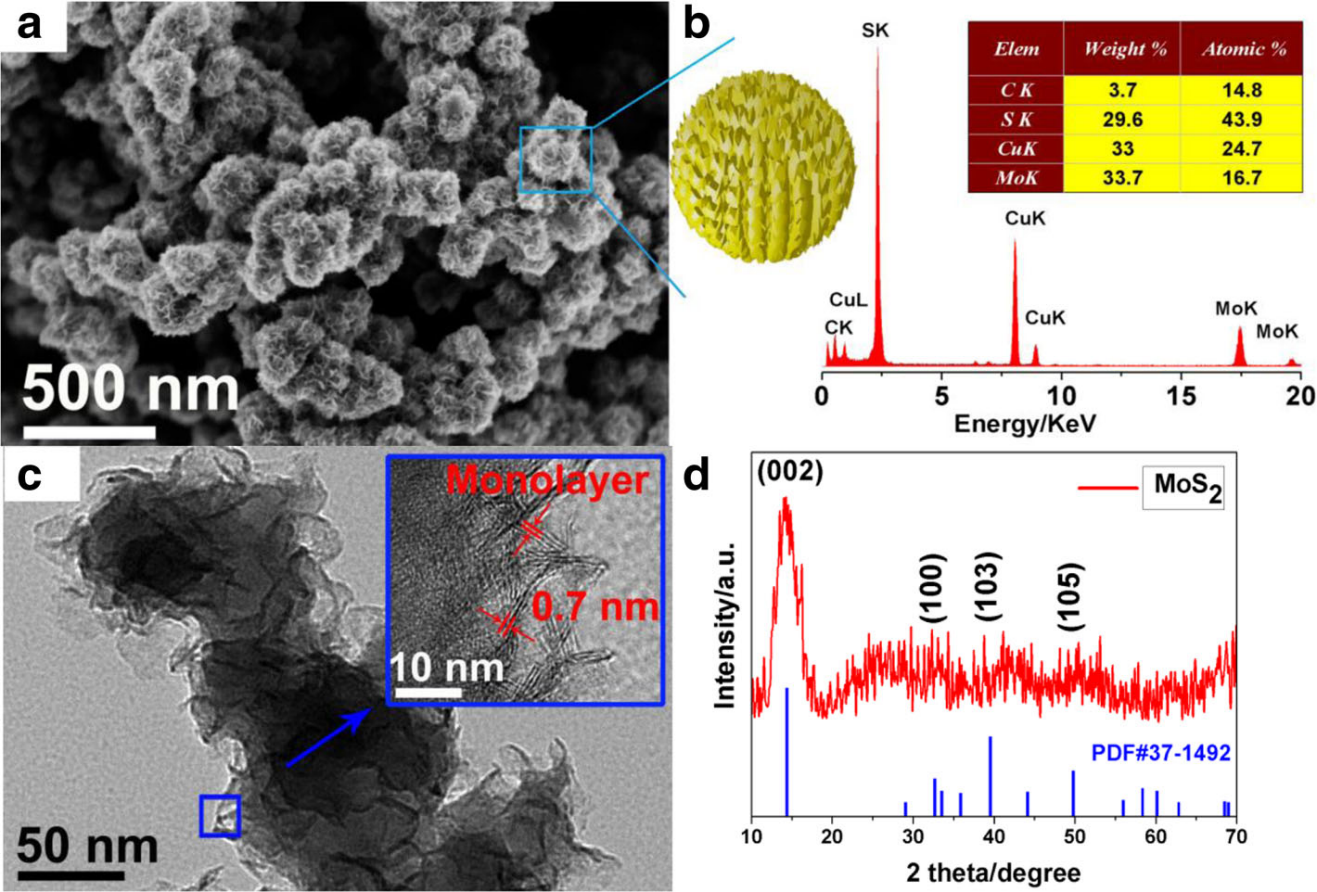
The SEM (**a**) and TEM (**c**) images of as-obtained naked MoS_2_. (*Inset* of Fig. 1c shows the HRTEM) and (**b**, **d**) are the EDX and XRD spectrum

The morphology of the as-obtained naked MoS_2_ nanoparticles are characterized by SEM and TEM. The representative SEM image is shown in Fig. 1a, in which the surface of flower-like MoS_2_ nanoparticles consisted of several or even single layers of ultra-thin MoS_2_ nanosheets. The average size of individual flower-like MoS_2_ nanoparticle is about 200 nm. As shown in Fig. 1c, high-resolution TEM image demonstrates that the thickness of MoS_2_ monolayer is ca. 0.7 nm, and the nanosheets consisted of about two to five layers, which could be indexed well with the theoretical thickness of MoS_2_ monolayer (S–Mo–S unit cell) [29, 30]. The EDX spectra of as-obtained MoS_2_ nanoparticles are shown in Fig. 1b, the elements of Mo, S, C, and Cu could be found. The structure of the naked MoS_2_ nanoparticles is further characterized by XRD (Fig. 1d), all the characteristic peaks can be assigned to the molybdenite-2H phase of MoS_2_ (JCPDS card no. 37–1492). The above results reveal the successful synthesis of MoS_2_ NPs.

**Fig. 2:**
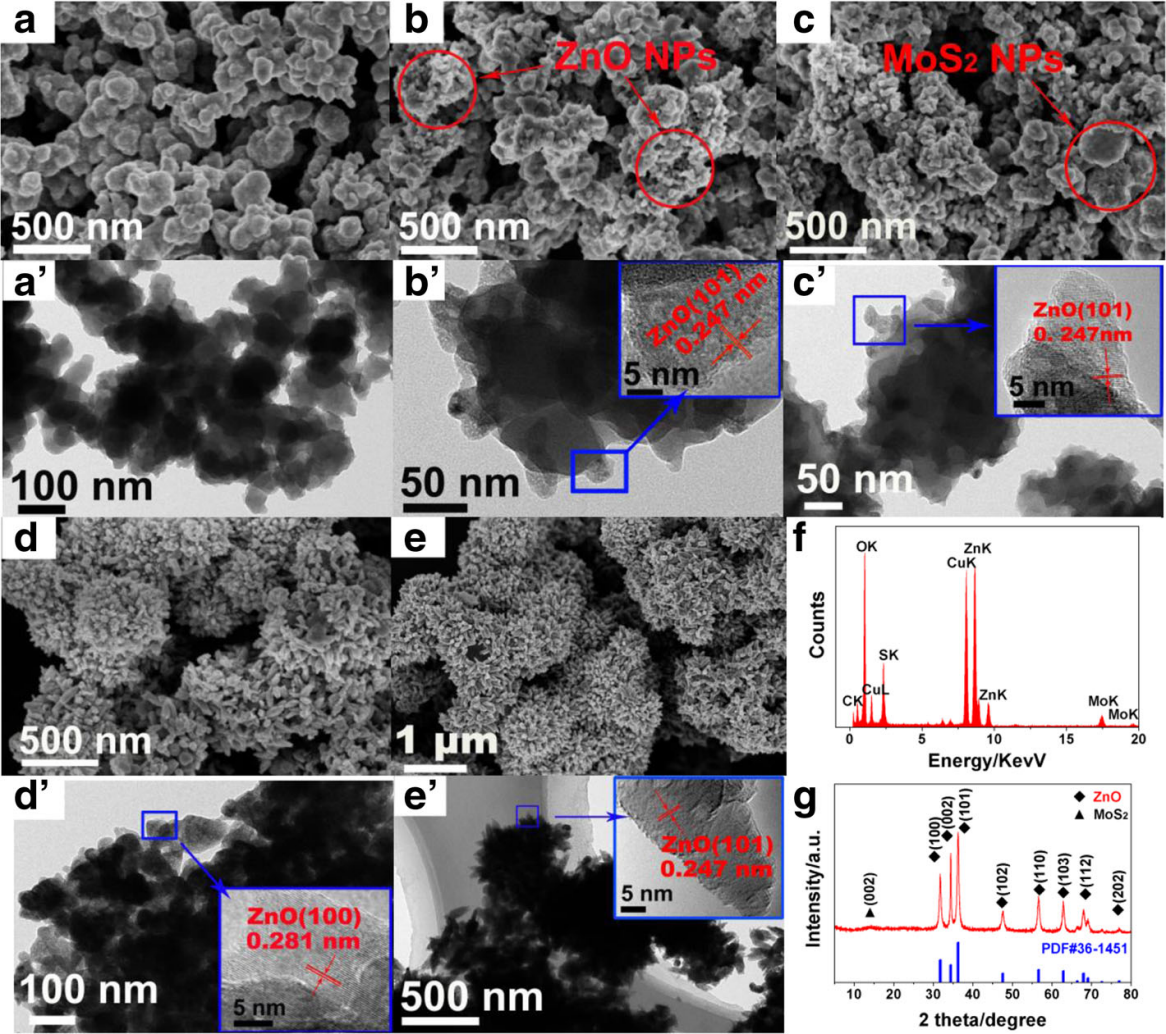
The SEM and TEM images of MoS_2_/ZnO with different mass proportion: (3:2) (**a**, **a**′), (3:4) (**b**, **b**′), (3:6) (**c**, **c**′), (3:10) (**d**, **d**′), and (3:20) (**e**, **e**′). *Inset* in (**b**′, **c**′, **d**′, **e**′) are the corresponding HRTEM images. The EDX (**f**) and XRD (**g**) spectra results from MoS_2_/ZnO (3:10)

Subsequently, the MoS_2_/ZnO heterostructures with different concentrations of zinc precursor are prepared. The detailed microstructural characterizations are presented in Fig. 2a–e, which show that the ZnO nanoparticles were successfully deposited on the surface of MoS_2_. Along with the increase of the concentration of Zn^2+^, both of the sizes and coverage area of ZnO nanoparticles are increased gradually. Compared with the uncoated MoS_2_ nanoparticles (Additional file 1: Figure S1), the morphology of MoS_2_/ZnO (3:2) shows no significant change because only a few ZnO nanoparticles are coated on the surface of MoS_2_ (Fig. 2a). When the Zn^2+^ is increased to 0.02 M, some small ZnO particles appeared (as marked by the red circle in Fig. 2b). When the Zn^2+^ concentration further increases to 0.03 M, most of MoS_2_ are coated by ZnO nanoparticles, the uncovered area of MoS_2_ was marked with red cycle in Fig. 2c. In addition, the size of ZnO nanoparticles grew much larger than 0.02 M. In Fig. 2d, the SEM image of MoS_2_/ZnO (3:10) shows that all the surface of MoS_2_ was covered with ZnO nanoparticles and the ZnO nanoparticles presented as triangle-shape. When the Zn^2+^ concentration elevated to 0.1 M, the ZnO particles with spindle-like structure that appeared on the surface of MoS_2_ nanosheets are observed. The TEM images of MoS_2_/ZnO are shown in Fig. 2a′–e′, displaying the gradual change of morphology during the ZnO coating process. From the corresponding high-resolution TEM images (inset), the (101) and (100) crystal planes of ZnO were observed, revealing the successful formation of ZnO nanoparticles on the surface of MoS_2_. The EDX and XRD techniques are employed for furtherly characterizing the elemental composition of as-obtained MoS_2_/ZnO heterostructure (MoS_2_/ZnO (3:10)). The EDX spectroscopy in Fig. 2f reveals the elements of Mo, S, Zn, C, and O. The XRD diffraction (Fig. 2g) peaks of MoS_2_/ZnO along with the JCPDS card for hexagonal ZnO (36–1451) and the diffraction signals of MoS_2_ are hardly observed, except for (002) plane (marked by “▲”), because the diffraction signals of ZnO is too strong and concealed the diffraction signals of MoS_2_. No other additional peaks corresponding to impurities are found, indicating the purity of the sample.

**Fig. 3:**
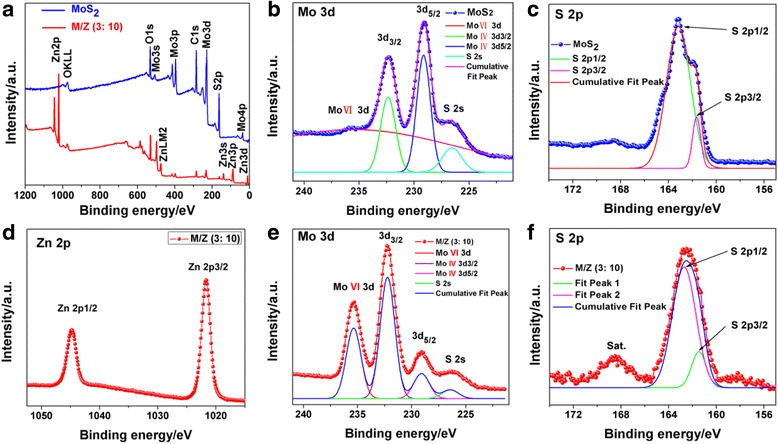
**a** The survey XPS spectra of naked MoS_2_ (*blue line*) and MoS_2_/ZnO heterostructure (MoS_2_/ZnO (3:10), *red line*). The high-resolution spectra of **b** Mo 3d, S 2s and **c** S 2p peaks for the MoS_2_ and the main satellite peaks of **d** Zn 2p, **e** Mo 3d, S 2s, and **f** S 2p for the MoS_2_/ZnO (3:10), respectively

The element valence in the naked MoS_2_ and MoS_2_/ZnO heterostructures (MoS_2_/ZnO (3:10)) are characterized by XPS. The full survey spectrum in Fig. 3a reveals the co-presence of Mo, S, O, C, and Zn elements. In the high-resolution XPS spectra of Mo 3d (Fig. 3b), two peaks are located at 232.4 and 229.2 eV, which could be attributed to Mo 3d 3/2 and Mo 3d 5/2 of Mo^4+^ in MoS_2_, respectively. The peak at 226.5 eV is indexed to S 2s. The peak positioned at the binding energy of 161.7 and 163.1 eV in S 2p spectra (Fig. 3c) are indexed to S^2−^ ions in the Mo–S bonding of the molybdenite-2H phase of MoS_2_, respectively. After coating with ZnO, the binding energies of 232.3, 229.1, 161.4, and 162.8 eV can be ascribed to Mo 3d 3/2, Mo 3d 5/2, S 2p 1/2, and S 2p 3/2, respectively (Fig. 3e, f). And the main peaks of Zn 2p at 1044.8 and 1021.7 eV are characteristic peaks of the Zn 2p 1/2 and Zn 2p 3/2 of ZnO, respectively (Fig. 3d). The asymmetric peaks at 235.4 eV could be indexed to Mo (VI) 3d, indicating the oxidation of a small amount of Mo [13, 31]. The TEM, XRD, and XPS results illustrate the successful preparation of MoS_2_/ZnO heterostructures.

**Fig. 4:**
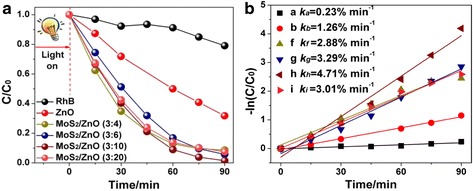
**a** The comparison degradation performance of MoS_2_/ZnO (3:4), MoS_2_/ZnO (3:6), MoS_2_/ZnO (3:10), MoS_2_/ZnO (3:20) heterostructures, and pure ZnO: the curves are normalized after the adsorption/desorption equilibrium in dark for 30 min. **b** The corresponding kinetic characters of photocatalytic degradation

The photodegradation performances of MoS_2_/ZnO heterostructures are investigated by the degradation of RhB dyes as a model experiment under illumination of simulated sunlight. Figure 4a shows the comparison of photocatalytic activity of bare control, pure ZnO nanoparticles, and as-obtained MoS_2_/ZnO heterostructures. All the curves are normalized after reaching the adsorption/desorption equilibrium. The results demonstrated that 68.3% of the RhB dyes can be degraded after 90 min light irradiation at the presence of pure ZnO. In contrast, MoS_2_/ZnO heterostructures show enhanced photocatalytic performance and more than 91.4% of the RhB can be degraded. Moreover, the photocatalytic properties show increasing tendency from MoS_2_/ZnO (3:4) to MoS_2_/ZnO (3:6) and reach the highest at MoS_2_/ZnO (3:10). However, the MoS_2_/ZnO (3:20) heterostructures show decreased photocatalytic abilities than MoS_2_/ZnO (3:10), which could be attributed to high mass percentage of ZnO (with poor photodegradation performance), and finally decreased the total degradation performance of MoS_2_/ZnO heterostructures. Generally, the reaction kinetics of photocatalytic degradation is followed by the pseudo first-order rate model: [6].

1 $$ - \ln \left( C/{C}_0\right) = k t $$

where *k* is the photodegradation rate. In Fig. 4b, the *k* value increased 3.7 times from 1.26 × 10^−2^ min^−1^ for pure ZnO nanoparticles to 4.71 × 10^−2^ min^−1^ for MoS_2_/ZnO (3:10) heterostructure. And the photodegradation rate of the MoS_2_/ZnO (3:4), MoS_2_/ZnO (3:6), and MoS_2_/ZnO (3:20) are 2.88 × 10^−2^ min^−1^, 3.29 × 10^−2^ min^−1^, and 3.01 × 10^−2^ min^−1^, respectively. The enhanced photodegradation activities of the as-obtained MoS_2_/ZnO heterostructures could be attributed to the *p-n* heterojunction that formed between MoS_2_ and ZnO at the contact interface, and the induced high separation rate of photogenerated electrons and holes.

**Fig. 5:**
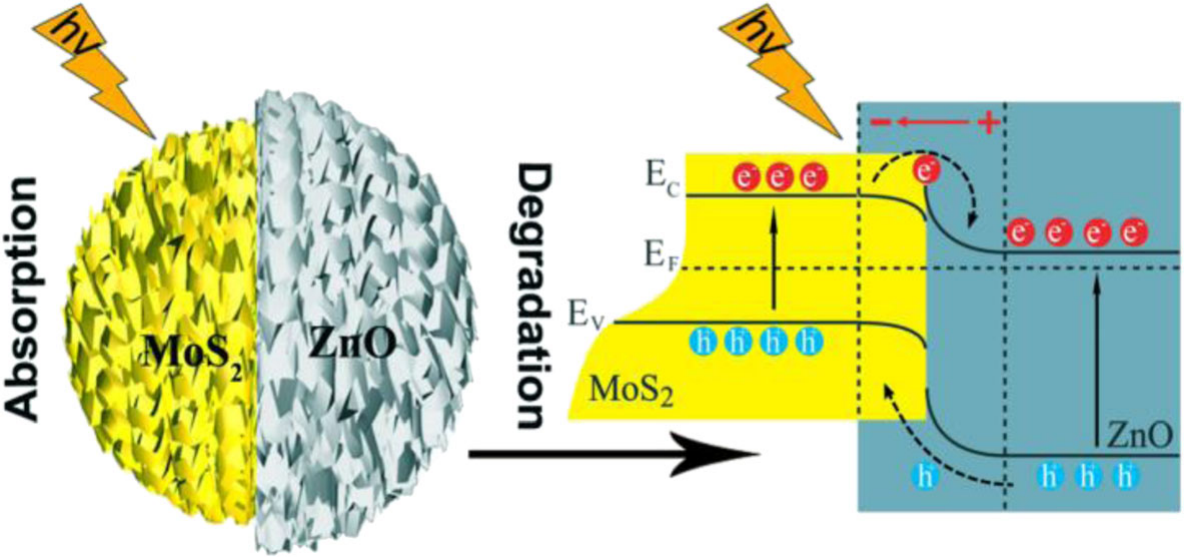
Schematic illustration of MoS_2_/ZnO heterostructures and the charge transfer process occurring between MoS_2_ and ZnO at the interface

The schematic illustrations of photocatalysis mechanisms are shown in Fig. 5, when the *n*-type semiconductor ZnO contact with the *p*-type semiconductor MoS_2_, the highest occupied conduction band (CB) and the valence band (VB) of ZnO situate below the energy band of MoS_2_ after reaching the same Fermi level due to the different work functions of the MoS_2_ (5.39 eV) [32, 33] and ZnO (4.7 eV) [29, 34]. Then, the photogenerated electrons at the CB of MoS_2_ can overcome the barrier and transfer to the CB of ZnO; meanwhile, the photogenerated holes will be in a position to transfer from the VB of ZnO to the VB of MoS_2_. Consequently, the photogenerated electron-hole pairs are separated efficiently at the contact interfaces of MoS_2_ and ZnO, thus, improving the photocatalytic abilities of the heterostructural catalysts finally.

**Fig. 6:**
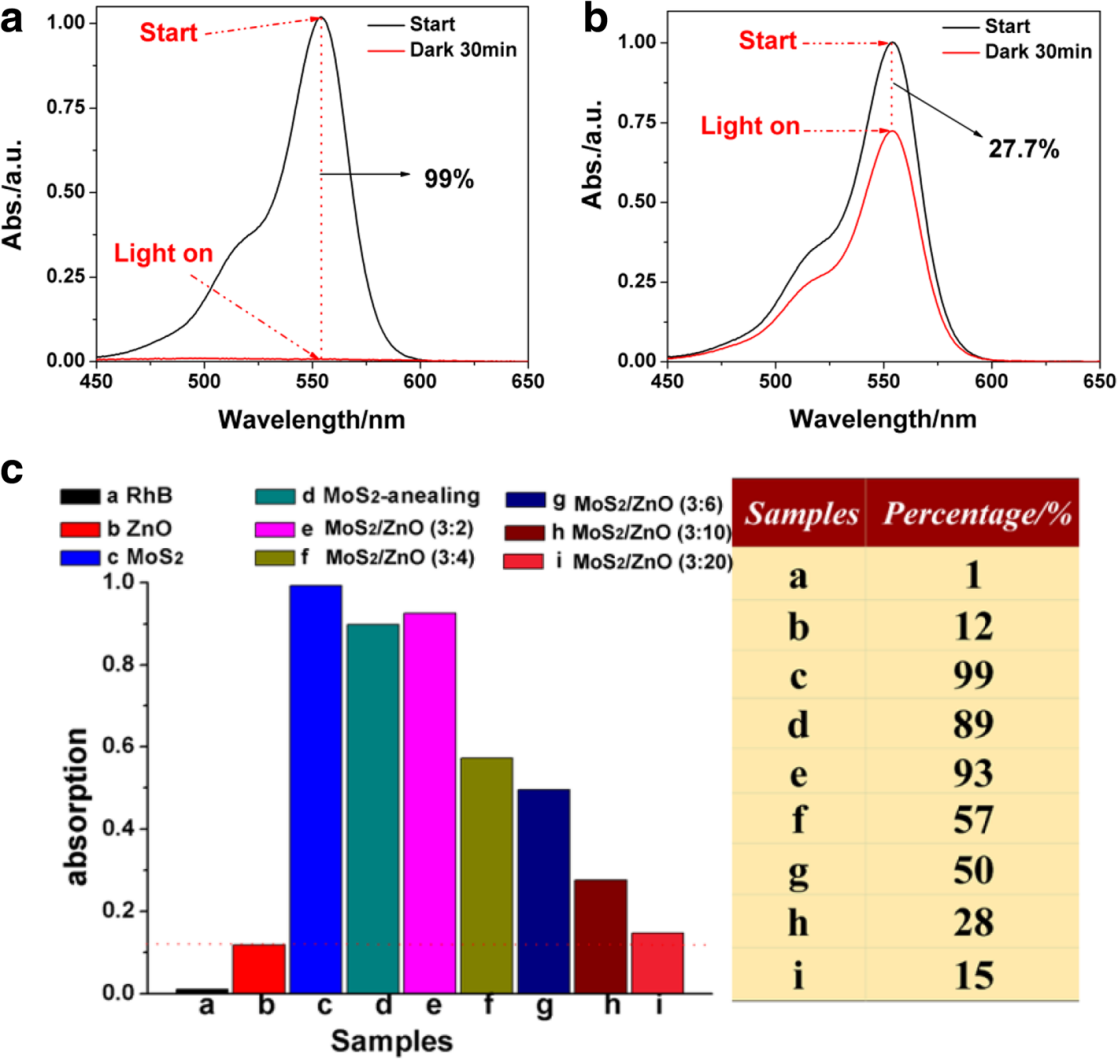
The UV-vis absorption spectra of naked MoS_2_ (**a**) and MoS_2_/ZnO (3:10) (**b**) heterostructures before light irradiation. **c** The comparison absorbance of all the samples and the corresponding absorbance percentage of RhB dyes: (*a*) blank RhB, (*b*) pure ZnO, (*c*) MoS_2_ before annealing, (*d*) MoS_2_ after annealing, (*e*) MoS_2_/ZnO (3:2), (*f*) MoS_2_/ZnO (3:4), (*g*) MoS_2_/ZnO (3:6), (*h*) MoS_2_/ZnO (3:10), and (*i*) MoS_2_/ZnO (3:20)

Meanwhile, when we measured the photocatalytic performance of MoS_2_/ZnO, it can be found that the surface adsorption plays an important influence in photocatalytic degradation process. It can be seen that 99% of RhB dyes can be absorbed by naked MoS_2_ nanoparticles before the light illumination (Fig. 6a), while 27.7% of RhB dyes are absorbed when using the MoS_2_/ZnO (3:10) heterostructures as the photocatalysts (Fig. 6b). Thus, we measured the absorption capacities of different samples in dark environment for 30 min. The comparative absorbance of all the samples was carried out in Fig. 6c. It can be seen that the MoS_2_/ZnO photocatalysts exhibit decreased adsorption capacity along with the increasing of the concentration of Zn precursor. Obviously, the ZnO coating can prevent the MoS_2_ to adsorb the dyes. The adsorption abilities of MoS_2_/ZnO photocatalysts are better than pure ZnO nanoparticles (12%, red dotted line in Fig. 6c). Furthermore, the naked MoS_2_ after annealing treatment show decreased adsorption abilities, only 89% of the RhB dyes were adsorbed. The reason may be resulted from the surface morphologic change of the flower-like MoS_2_ nanoparticles (Additional file 1: Figure S1) that decreases the adsorption sites. The corresponding Brunauer-Emmett-Teller specific surface areas (*S*_*BET*_) of MoS_2_ nanoparticles before and after annealing were investigated by nitrogen adsorption (Beishide, 3H-2000PS2). In contrast, the *S*_*BET*_ for flower-like MoS_2_ nanoparticles is about 54.7 m^2^/g, which is much larger than that of MoS_2_ nanoparticles after annealing treatment with a surface area of 13.2 m^2^/g (Additional file 1: Figure S2). The reason was largely attributed to the morphologic change of MoS_2_ nanoparticles, resulting in the decrease of the adsorption capacities to MG dyes finally.

**Fig. 7:**
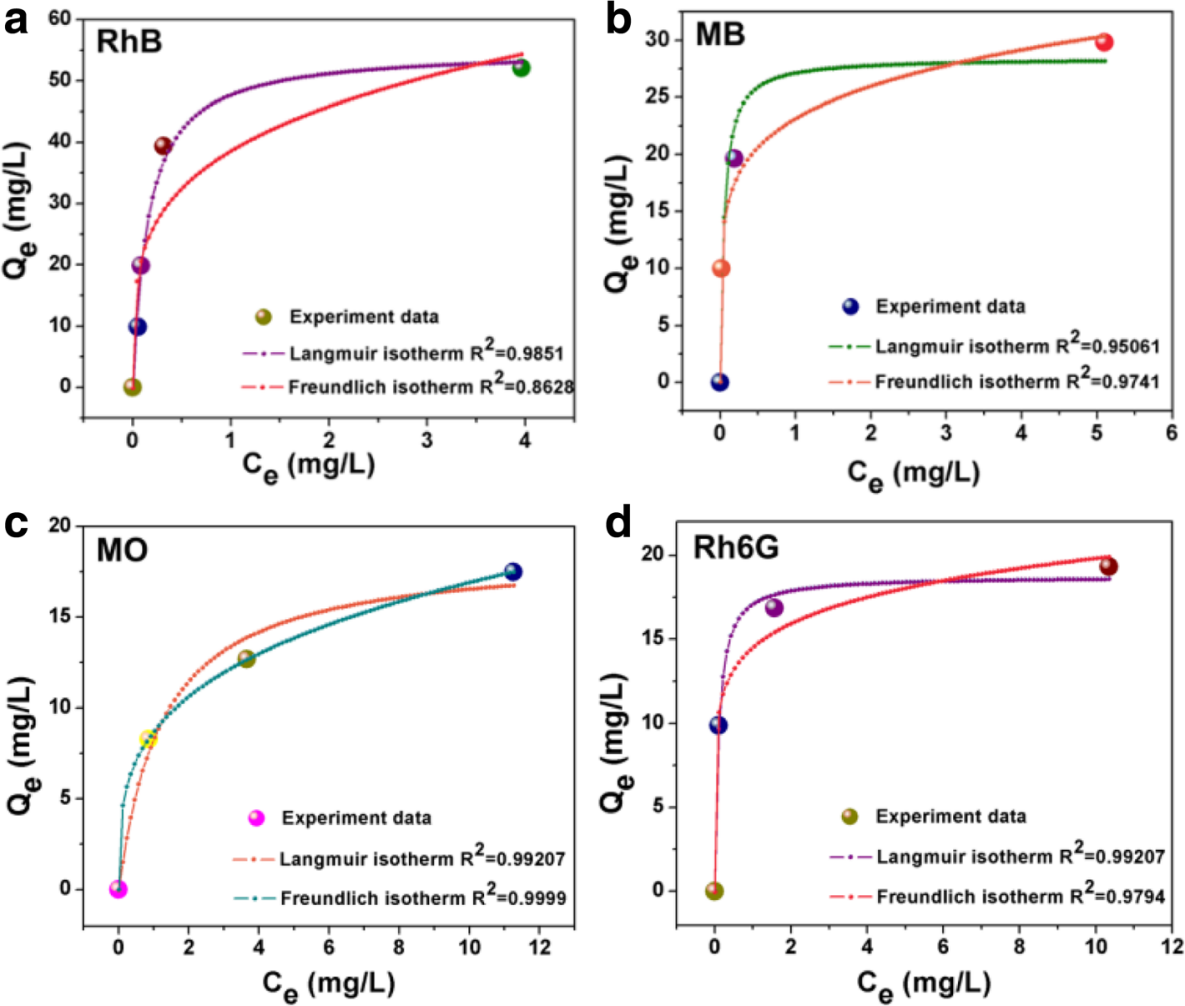
The adsorption isotherms for **a** RhB, **b** MB, **c** MO, and **d** Rh6G on the as-obtained naked MoS_2_ nanoparticles

The selective adsorption to organic dyes is also a critical factor for the photocatalytic performance of catalyst. As the main contributor to the adsorption capacity of the MoS_2_/ZnO heterostructures, the adsorptive capacity of the naked MoS_2_ was further measured with different organic dyes, including RhB, Rh6G, MB, and MO. The as-selective dyes are very stable in routine environment. In addition, different concentrations of organic dyes (5, 10, and 20 mg/L) are tested. Additional file 1: Figure S3 displays the relationship between the adsorption time and concentrations. It is found that the adsorption process can be finished in 5 min, and there is no continued adsorption with prolonging adsorption time. The experiment results are in agreement with the report that the adsorption equilibrium can be reached in a relatively quick process of about 5 min [30]. The adsorption capacity is calculated based on the experimental dates and according to the mass balance relationship: [35].

2 $$ {Q}_{eq}=\left({C}_0-{C}_{eq}\Big) V/ m\right) $$

where *Q*_*eq*_ is the amount of dye adsorbed onto the naked MoS_2_, and *C*_*0*_ and *C*_*eq*_ are the initial and equilibrated dyes concentrations, respectively. *V* is the volume of solution, and *m* is the mass of the adsorbent. Figure 7 shows the isotherms for dyes adsorption on the as-obtained naked MoS_2_ nanoparticles. All the adsorption isotherms that fitted to the experimental dates are modeled using the Langmuir and Freundlich isotherm (Table 1) [2]. The theoretical Langmuir and Freundlich isothermal parameters can be represented by the following Eqs. 3 and 4 [36, 37].

**Table 1:** Equilibrium Parameters for the Adsorption of RhB, MB, MO and Rh6G onto naked MoS_2_

	Langmuir Fit			Freundlich Fit		
	$$ {Q}_e=\frac{Q_{\max }{K}_L{C}_e}{1+{K}_L{C}_e} $$			$$ {Q}_e={K}_F{C_e}^{\raisebox{1ex}{$1$}\!\left/ \!\raisebox{-1ex}{$\mathrm{n}$}\right.} $$		
Dyes	*Q*_max_ (mg/g)	*K*_*L*_(L/mg)	*R* ^2^	*K*_*F*_ (mg^1–1/*n*^ L^1/*n*^ g^−1^)	*n*	*R* ^2^
RhB	55.22	6.43	0.98506	38.65	4.03	0.8628
MB	28.43	20.15	0.95061	23.10	5.99	0.9741
MO	18.65	0.80	0.97768	8.70	3.46	0.9999
Rh6G	18.79	10.25	0.99207	14.55	7.39	0.9794

*Q*_*e*_ (mg/g) is the amount of dyes adsorbed onto the adsorbent at equilibrium, *Q*_*max*_ (mg/g) is the maximum amount of adsorption, *K*_*L*_ (L/mg) is the Langmuir adsorption equilibrium constant, *K*_*F*_ (mg^1–1/*n*^ L^1/*n*^ g^−1^) is the Freundlich constant representing the adsorption capacity, *n* (dimensionless) is the constant depicting the adsorption intensity

3 $$ {Q}_{eq}=\frac{Q_{\max }{K}_L{C}_{eq}}{1+{K}_L{C}_{eq}} $$

4 $$ {Q}_{eq}={K}_F{C_{eq}}^{\raisebox{1ex}{$1$}\!\left/ \!\raisebox{-1ex}{$ n$}\right.} $$

*Q*_*e*_ is the amount of dyes adsorbed onto the adsorbent at equilibrium, *Q*_*max*_ is the maximum amount of adsorption, *K*_*L*_ is the Langmuir adsorption equilibrium constant, *K*_*F*_ is the Freundlich constant representing the adsorption capacity, and *n* is the constant depicting the adsorption intensity. The maximum adsorption capacities calculated from the experimental date for RhB, MB, MO, and Rh6G are 55.2, 28.4, 18.6, and 18.8 mg/g, respectively. The results reveal that the naked MoS_2_ has a non-selective adsorption to the organic dyes with perfect adsorption capacities. From the nonlinear-curve-fitting results (*R* ^2^), we can see that the Langmuir isotherm present well with the experimental dates than that of Freundlich isotherm for RhB (0.9851 > 0.8628) and Rh6G (0.9921 > 0.9794), while the adsorption value is concurred with agreement with the Freundlich model than Langmuir model from the regression coefficient *R* ^*2*^ of MB (0.9506 < 0.9741) and MO (0.9921 < 0.9999). For the comparison, the absorption capacity of pure ZnO particles is tested. The corresponding effect of contact time and concentration is shown in Additional file 1: Figure S4a. The maximum adsorption amount is only 2.4 mg/g (*R* ^2^ = 0.9985) from the Langmuir model fitting result in the Additional file 1: Figure S4b.

From the adsorption isotherm studies, we can draw the conclusion that the pure ZnO nanoparticles has a poor adsorption to RhB dyes, which decreased the adsorption capacity of MoS_2_/ZnO heterostructures. MoS_2_ nanoparticles act as the main adsorption substrate in the heterostructural system and show perfect and non-selective adsorption capacity to the organic dyes. The Langmuir and Freundlich modeled results can be confirmed that the organic dyes inhibited monolayer adsorption on the surface of MoS_2_ nanoparticles and further indicated that the homogeneous nature of MoS_2_ surface has a same adsorption capacity of all the surfaces [36]. Moreover, the adsorption intensity (*1*/*n*) gives an indication of the favorability of adsorption. When the *n* > 1 (or 10 > 1/*n* > 1), it represents favorable adsorption condition [38, 39]. The calculated values of *n* are listed in Table 1, confirming the favorable adsorption condition of dyes on the surface of MoS_2_ nanoparticles.

## Conclusions

In summary, the naked flower-like MoS_2_ nanoparticles are synthesized successfully via a hydrothermal method. The MoS_2_/ZnO heterogeneous photocatalysts are synthesized and formed as *p-n* heterojunction. The coating of ZnO nanoparticles shows positive promotion to the photodegrading properties while negative effect on the adsorption capacity of the MoS_2_/ZnO heterostructures. The MoS_2_/ZnO (3:10) heterostructures showed 3.7 times than pure ZnO for the decomposition of RhB dyes. The adsorption property of naked MoS_2_ was further investigated with RhB, MB, MO, and Rh6G. The adsorption relationship was described with Langmuir and Freundlich isotherm, showing that the adsorption of dye molecules on the surface of MoS_2_ is monolayer adsorption and it is favorable for the adsorption of organic dyes in our study. We envision that the MoS_2_/ZnO photocatalysts will become a potential candidate in the field of environmental remediation.

## Additional file

Additional file 1: Figure S1. Supporting information is available online from the Wiley Online Library or from the author. (DOCX 1281 kb)
