# Supplementary material for: Zinc Oxide Coating Effect for the Dye Removal and Photocatalytic Mechanisms of Flower-Like MoS2 Nanoparticles
Source: Nanoscale Res Lett. 2017 Mar 23;12:221. doi: 10.1186/s11671-017-2005-0 (PMC5364121; doi:10.1186/s11671-017-2005-0)
Supplement: Additional file 1: Figure S1. — Supporting information is available online from the Wiley Online Library or from the author. (DOCX 1281 kb) [file 11671_2017_2005_MOESM1_ESM.docx]

Zinc Oxide Coating Effect for the Dye Removal and Photocatalytic Mechanisms of Flower-like MoS_2_ Nanoparticles

*Qingyong Tian^1^, Wei Wu^1,^ ^2^[[1]](#footnote-1)^*^, Shuanglei Yang^1^, Jun Liu^1^, Weijing Yao^1^, Feng Ren^1^, Changzhong Jiang^1^**

^1^ School of Printing and Packaging and School of Physics and Technology, Wuhan University, Wuhan 430072, P. R. China.

^2^ Suzhou Research Institute of Wuhan University, Suzhou 215000, P. R. China.


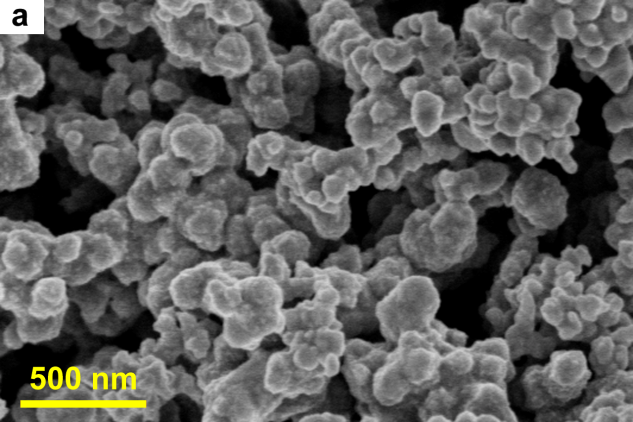

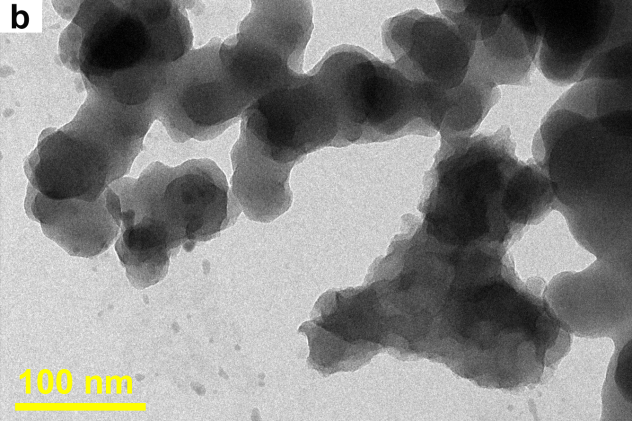


**Figure S1** The SEM (a) and TEM (b) image of as-obtained naked MoS_2_ after annealing at 200 ºC for 2 hours


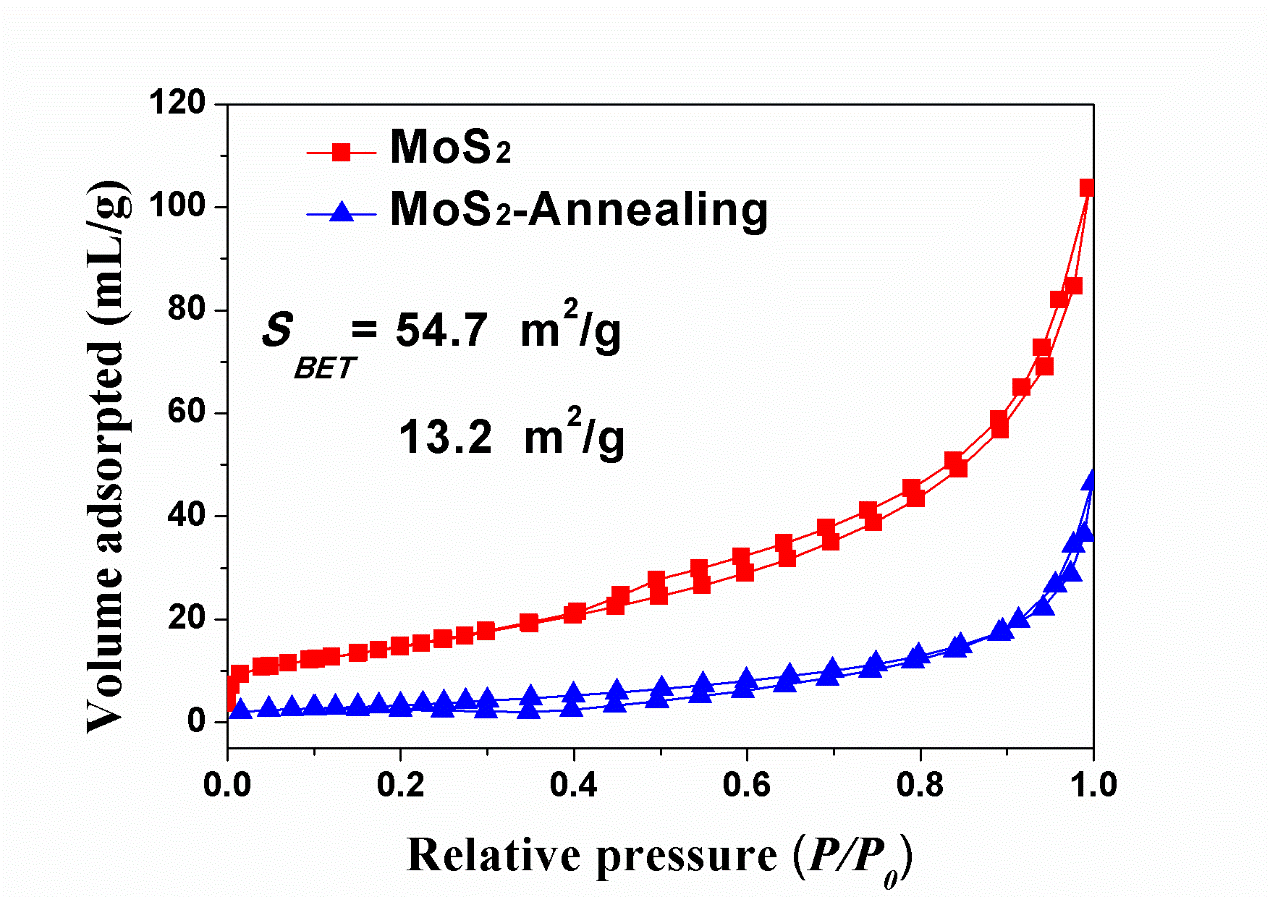


**Figure S2** Nitrogen adsorption-desorption isotherms of the MoS_2_ (red) and MoS_2_-annealing (blue) samples


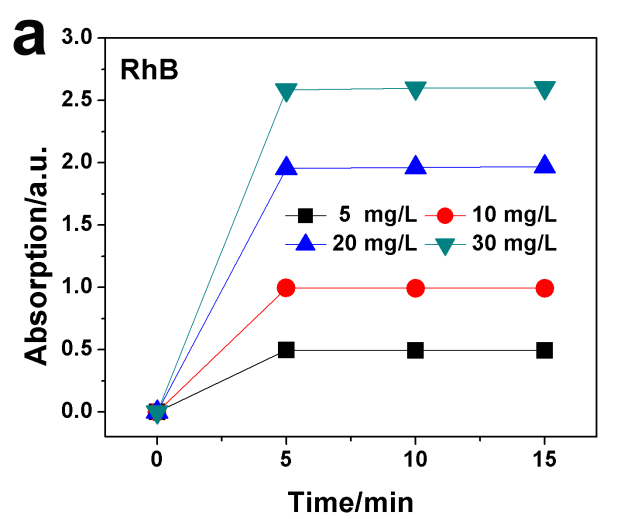

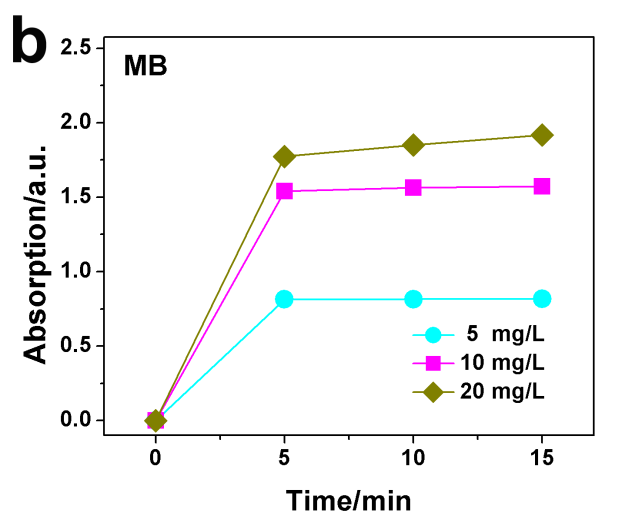

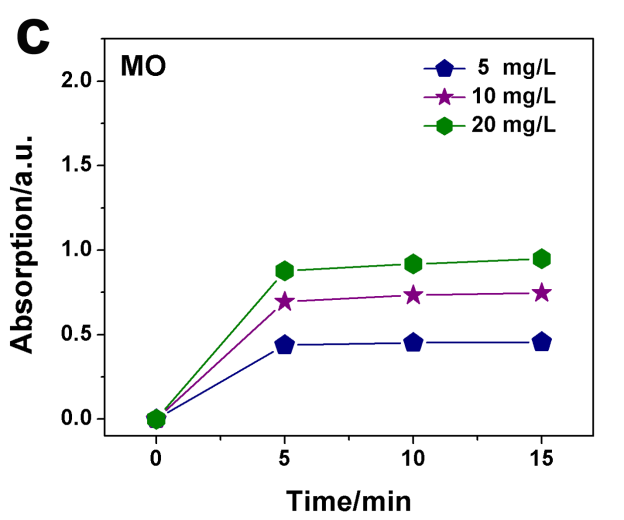

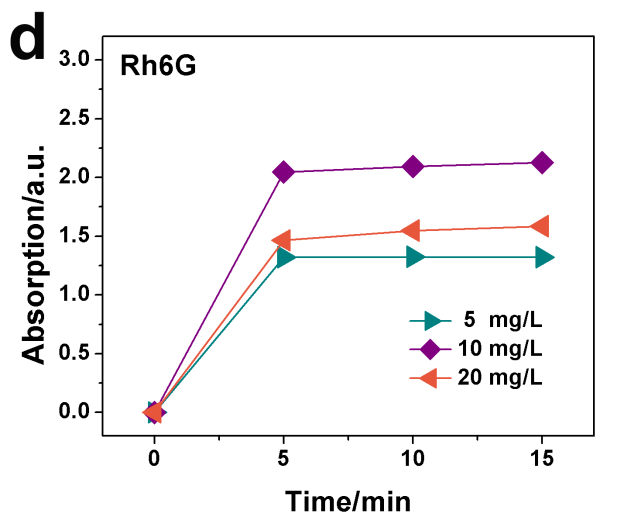


**Figure S3** The effect of contact time and concentration on adsorption of (a) RhB, (b) MB, (c) MO and (d) Rh6G onto naked MoS_2_.


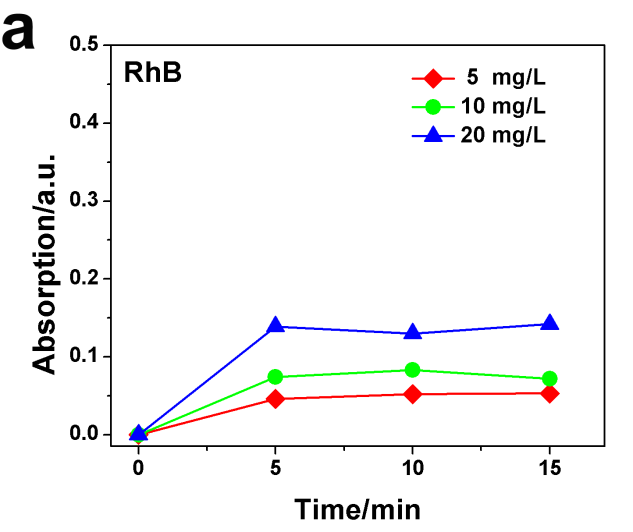

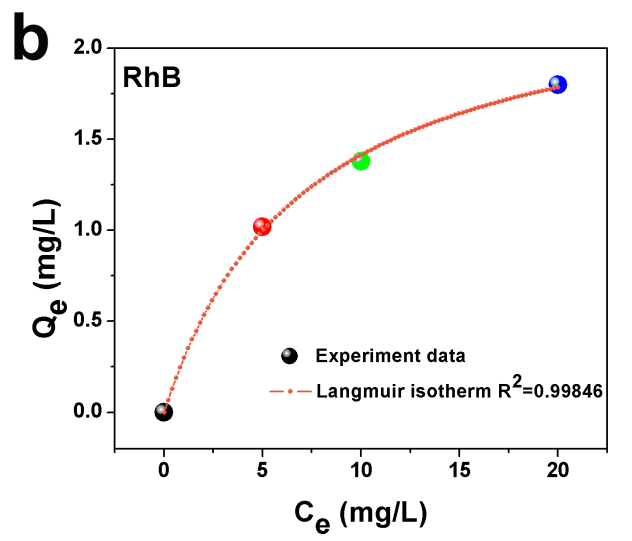


**Figure S4** The effect of contact time and concentration on adsorption of RhB (a) and the adsorption isotherms (b) on pure ZnO nanoparticles.

1. *To whom correspondence should be addressed. Tel: +86-27-68778529. Fax: +86-27-68778433. E-mail: [weiwu@whu.edu.cn](mailto:weiwu@whu.edu.cn) (W. Wu) and [czjiang@whu.edu.cn](mailto:czjiang@whu.edu.cn) (C. Jiang). [↑](#footnote-ref-1)
